# Social Skills Intervention Participation and Associated Improvements in Executive Function Performance

**DOI:** 10.1155/2017/5843851

**Published:** 2017-09-17

**Authors:** Shawn E. Christ, Janine P. Stichter, Karen V. O'Connor, Kimberly Bodner, Amanda J. Moffitt, Melissa J. Herzog

**Affiliations:** ^1^Department of Psychological Sciences, University of Missouri, Columbia, MO, USA; ^2^Thompson Center for Autism & Neurodevelopmental Disorders, University of Missouri, Columbia, MO, USA; ^3^Department of Special Education, University of Missouri, Columbia, MO, USA

## Abstract

Autism spectrum disorder (ASD) is a neurodevelopmental disorder characterized by impairments in social communication. It has been postulated that such difficulties are related to disruptions in underlying cognitive processes such as executive function. The present study examined potential changes in executive function performance associated with participation in the Social Competence Intervention (SCI) program, a short-term intervention designed to improve social competence in adolescents with ASD. Laboratory behavioral performance measures were used to separately evaluate potential intervention-related changes in individual executive function component processes (i.e., working memory, inhibitory control, and cognitive flexibility) in a sample of 22 adolescents with ASD both before and after intervention. For comparison purposes, a demographically matched sample of 14 individuals without ASD was assessed at identical time intervals. Intervention-related improvements were observed on the working memory task, with gains evident in spatial working memory and, to a slightly lesser degree, verbal working memory. Significant improvements were also found for a working memory-related aspect of the task switching test (i.e., mixing costs). Taken together, these findings provide preliminary support for the hypothesis that participation in the SCI program is accompanied by changes in underlying neurocognitive processes such as working memory.

## 1. Introduction

Autism Spectrum Disorder (ASD) is a neurodevelopmental disorder characterized by persistent impairments in social communication and social interactions as well as the presence of restricted/repetitive patterns of behavior [1]. The core impairments of ASD often manifest as difficulties in basic social and communicative behavior (e.g., eye contact, intonation, and facial expressions) as well as rigidity relating to routines and pervasive preoccupations [2]. Difficulties in social interaction skills often include a lack of social reciprocity, lack of nonverbal behaviors/gestures, and difficulty maintaining peer relationships [1, 3]. Although the onset and diagnosis of ASD most often occur in early childhood, the impairments are present across the lifespan [4] and may be particularly challenging during the adolescent period.

Research has suggested that the significant social-communicative impairments in individuals with ASD are directly related to negative long-term outcomes [5, 6]. Social communication impairments may manifest differently based on an individual's overall level of intellectual functioning. For example, social-communicative difficulties observed in individuals with high functioning autism (HFA; i.e., individuals average to above average cognitive abilities) often include the failure to recognize social cues and contexts, and therefore students struggle with appropriate conversational skills and interpreting nonverbal behaviors of others [7]. Specifically, the social impairments for individuals with HFA include impairments in the use of nonverbal behaviors, developmentally inappropriate peer relationships, failure to seek out others for enjoyment, and poor social-emotional reciprocity [1, 8]. These impairments can manifest both from a lack of knowledge of social skills and from difficulties in demonstrating these skills in the required contexts [9].

### 1.1. Social Challenges and Social Competence Intervention

Social difficulties experienced by individuals with ASD appear to be related to disruptions in various social cognitive processes. Prominently discussed constructs underlying the social challenges for individuals with autism include emotion recognition [10, 11], theory of mind [12, 13], and executive functioning [14, 15]. Other contributing constructs include social motivation [16, 17], social reciprocity [18], and social problem solving [19, 20]. Importantly, these constructs are not orthogonal; rather, they appear to be interrelated components to an integrated whole (for brief review, see [21]).

The Social Competence Intervention-Adolescent (SCI-A; [22, 23]) is based on cognitive behavioral intervention and applied behavior analytic principles and targets EF, theory of mind, and emotion recognition as key constructs in addressing social competence impairments. Although other researchers have investigated the potential role of EF, theory of mind, and emotion recognition in existing intervention programs [21], SCI-A was developed specifically with these target constructs in mind. The SCI curriculum is designed to challenge thinking patterns related to these underlying constructs and includes the following key components: use of metacognitive strategies, self-monitoring and self-regulation, and exposure and response situations [21, 24]. The curricular units (recognizing facial expressions, sharing ideas, turn taking in conversations, recognizing feelings and emotions of self and others, and problem solving) are presented in a scaffolded fashion, with each new unit building upon the content and skills of the previous ones (for additional description of the SCI program, see [22, 23]). Maintenance of learned skills is reinforced throughout the curriculum by the use of repetition, integration, and feedback as new skills are added. The curricular units utilize a combination of didactic instruction, behavior modeling, rehearsal, and in vivo practice with other intervention participants as a means to teach and/or modify social behavior specific to a student's needs.

### 1.2. Executive Function and Its Role in Social Competence

Although a precise definition of EF remains elusive, EF can be generally conceptualized as referring to a set of higher-order cognitive processes that allow for the flexible modification of thought and behavior in response to changing cognitive or environmental contexts [25]. It encompasses abilities such as planning, strategy use, organization, working memory, inhibitory control, and cognitive flexibility. These abilities are considered “executive” in that they require the integration and processing of information from a wide range of internal and external sources. Empirical evidence [26] suggests that at least three core component processes may comprise EF: updating (working memory), inhibition (inhibitory control), and shifting (cognitive flexibility).

Working memory is broadly defined as the active maintenance and manipulation of a limited amount of information over a short period of time [27, 28]. Inhibitory control refers to the ability to suppress the activation, processing, or expression of information that would otherwise interfere with efficient attainment of a cognitive or behavioral goal [29, 30]. Shifting, also called cognitive flexibility or rule shifting, refers to switching between tasks or mental sets in response to changing task demands [31]. While distinguishable, these three component processes are not necessarily orthogonal and, in fact, the interplay among them provides the foundation for more complex executive abilities such as planning, strategy use, and organization.

Previous research indicates that EF represents an area of particular weakness for individuals with ASD even after accounting for comorbid conditions such as ADHD [32, 33]. Meta-analytical reviews of the existing literature point to cognitive flexibility and working memory as areas of consistent weakness in individuals with ASD as compared to healthy individuals without ASD (for reviews, see [34, 35]). Findings on inhibitory control in ASD, however, have been much more mixed. Whereas a number of studies have reported significant impairments on measures of inhibitory control in individuals with ASD [36–39], others have failed to find a difference between individuals with ASD and their control counterparts [40–42]. Literature reviews on ASD and inhibitory control have also come to differing conclusions [35, 43].

Each of the aforementioned EF component processes can be hypothesized to play an important role in proficient social competence. For example, working memory allows one to follow the flow of a conversation/interaction while at the same time preparing his/her own contribution to the conversation [44]. Cognitive flexibility is critical in that moment-to-moment changes in conversational topic or context may necessitate a shift in which social cues are relevant and the appropriate response to such cues [45]. Lastly, inhibitory control allows one to ignore irrelevant cues/stimuli so that socially relevant information may be processed more efficiently [37].

Although a number of studies have examined potential group-related differences in EF and social competence within the same study (e.g., ASD group has both poorer EF and poorer social communication abilities compared to non-ASD group), few studies have reported analysis of within-group covariability of the two factors (i.e., the correlation between EF and social competence within a given ASD sample). For the handful of studies that have done so, the results have been mixed. Some studies have found significant correlations between measures of EF and social competence [46–49], but others have failed to find any such evidence [50, 51]. In a recent randomized clinical trial, Kenworthy et al. [52] found similar gains in social competence for individuals with ASD who participated in an executive function-focused intervention compared to those who participated in a more general social skills-focused intervention.

Developmental research with younger children provides additional support for the interrelationship between EF and social competence. Findings from several studies suggest that early EF abilities are correlated with later theory of mind ability [53–56]. Also, in a more recent study, Pellicano [57] found that individual differences in early EF performance at age 5–7 years predicted social communication ability (as well as extent of repetitive behaviors/restricted interest) in children three years later.

Previous research [22, 23] supports the effectiveness of the SCI-A program in improvements in parent reports of social competence behaviors and characteristics of adolescents with HFA. In addition, pre- to postintervention improvements in EF as assessed by measures such as the Behavior Rating Inventory of Executive Function (BRIEF; [58]) and the Test of Problem Solving-3 (TOPS-3; [59]) were also found in these studies. The BRIEF is a parent- or teacher-report measure designed to assess behavioral manifestations of EF problems in daily life. The TOPS-3 is a performance-based measure in which participants are shown photographic or written scenarios and asked questions designed to assess the participants' interpretation of what factors may have contributed to the scenario and potential ways to solve the situation. Both the BRIEF and TOPS-3 focus primarily on complex manifestations of EF (e.g., everyday problem solving), which rely on a confluence of working memory, inhibitory control, and cognitive flexibility. Little is currently known regarding the relationship between Social Competence Intervention and potential changes in individual EF component processes.

### 1.3. The Current Study

As noted above, there is growing evidence [22, 23] of potential improvements in EF for adolescents who participated in the SCI-A intervention. To date, however, these studies have relied primarily on measures (e.g., the BRIEF and TOPS) that capture EF in broad-brush strokes. The current study builds on these results by examining the nature of such improvements via laboratory behavioral performance measures to evaluate potential intervention-related changes in individual EF component processes in a sample of adolescents with HFA who participated in SCI-A. A demographically matched sample of adolescents without ASD was assessed at identical time intervals for comparison purposes.

Digit span and spatial span tests were used to assess verbal and nonverbal working memory, respectively. Cognitive flexibility was assessed using a computerized switching task in which participants performed a visual matching task in which the response rule (i.e., whether to match the stimuli based on shape or color) was pseudo-randomly switched from trial to trial. In terms of inhibitory control, we focused on the ability to filter and resist interference from visual distractors, an aspect of inhibitory control that is known to be particularly affected in ASD [36, 37].

Based on previous research, we hypothesize that the ASD group will perform more poorly than the non-ASD group at baseline on all of the EF tests. However, we also hypothesize that improvements in EF performance will be observed in the ASD group with participation in the SCI intervention, thus decreasing the magnitude of group differences observed at follow-up.

## 2. Methods

### 2.1. Participants and Setting

Data is presented for a sample of 22 individuals (all male) with HFA ranging in age from 10.8 to 14.7 years (*M* = 12.3, SD = 1.14) who completed the SCI-A program. Additional demographic information on the sample is included in Table 1.

The program was delivered at a university-affiliated interdisciplinary diagnostic and outpatient treatment center for ASD and neurodevelopmental disorders in the Midwest. In total, SCI-A included 20 hours of group intervention delivered in 1-hour lessons twice weekly for 10 weeks. The groups met during academic semesters after school hours in a classroom setting within the autism center. Master's level implementers with specific training in special education with ASD specializations led all current sessions. Cohorts were restricted to no more than seven participants each (range = 4–7 participants). As noted earlier, both content and instruction were scaffolded over time with new skills and practice opportunities layered upon each other as the curriculum progressed.  Table 2 provides a description of how the SCI-A curricular units are conceptually linked to different EF component processes.

As noted previously, the SCI-A program designed for adolescents with HFA with a specific, predetermined set of characteristics (phenotype) related to their age, level of functioning, and general education placement. To be enrolled in the SCI-A program, participants had to meet the following criteria [22]: (a) age 11–14 years; (b) a clinical or medical diagnosis of an ASD; (c) a full scale IQ score above 75; (d) scores on the Autism Diagnostic Observation Schedule (ADOS; [60]) and/or Autism Diagnostic Interview-Revised (ADI-R; [61]) that met or exceeded clinically designated cutoffs; and (e) access to typically developing peers without ASD for at least part of their day (e.g., via general education classrooms). These are similar to the criteria utilized by Solomon et al. [21].

The current participants represent a subset of individuals across four semesters that were concurrently enrolled in a larger efficacy study of the SCI-A program [22]. Within these targeted four semesters, some participants were excluded from the present analysis for various reasons. Five were excluded due to excessive absences from the SCI-A program (>20% of sessions missed; attendance for the remaining participants were very high, with an average of <5% of sessions missed). Three participants were excluded due to compliance issues at pre/posttesting.

For comparison purposes, data was also reported for an age- and gender-matched comparison sample of 14 neurologically uncompromised individuals (all male) without ASD. Participants in the non-ASD comparison group ranged in age from 11.0 to 14.5 years (*M* = 12.8; SD = 0.91). They were recruited from the Columbia, Missouri community. Prior to enrollment, the parents of potential participants were asked to complete an extensive questionnaire detailing past developmental and medical history. Individuals with significant medical and/or psychiatric history were excluded. Additional demographic information on the sample is included in Table 1.

The non-ASD comparison group was evaluated at an equivalent time interval (12–14 weeks) to the ASD intervention group; however, they did not receive the SCI intervention. The ASD and non-ASD groups did not differ significantly in terms of age [*t*(34) = 1.43, *p* = .16] or FSIQ [*M*_ASD_ = 100.5; *M*_TYP_ = 103.4; *t*(34) < 1, *p* = .45].

### 2.2. Procedure

The present study was approved by the University of Missouri-Columbia Internal Review Board and completed in accordance with the Helsinki Declaration. Informed consent and assent were obtained for all participants in the present study.

Baseline evaluations were conducted with intervention participants within a 2-week window prior to beginning the SCI-A program. The SCI-A program was then delivered over a 10-week period twice a week for an hour within a clinic-based classroom setting. Participants completed postintervention evaluation within two weeks following completion of the program. The non-ASD comparison group did not receive the intervention but had baseline and postevaluation at an equivalent time interval (12–14 weeks) to the intervention group. All measures reported below were administered at both baseline and posttest.

All EF tasks were administered in a small, quiet room with sufficient overhead lighting. For the computerized tests, reaction time (RT) and error rate were recorded for each condition. Children used both hands to respond during the inhibitory control task (i.e., left hand to the left button; right hand to the right button). For the cognitive flexibility/switching task, participants used their dominant hand to respond. The order of task administration was varied randomly across participants.

As evidenced by the relatively low error rates observed, none of the children exhibited difficulties in understanding task instructions or feedback for any of the tasks. In addition, to further ensure that the children had time to become comfortable with the computer tasks, each task included a block of practice trials that was administered prior to data collection. [Note that the Digit Span and Spatial Span working memory tasks were administered as per their standardized instructions, which does not include “practice trials” per se.]

### 2.3. Measures

#### 2.3.1. Working Memory

The Digit Span and Spatial Span subtests of the Wechsler Intelligence Scale for Children – Fourth Edition Integrated (WISC-IV Integrated; [62]) were administered to assess verbal and nonverbal working memory, respectively. The Digit Span subtest has two components: Digits Forward and Digits Backwards. In the Digits Forward component, the participant must repeat back a series of auditorily-presented digits in the same order as they were presented. In the Digits Backwards component, the participant again receives a series of auditorily presented digits, but he/she must now repeat them back in reverse order. In both components, the length of the sequence increases as the participant responds correctly.

The Spatial Span subtest was designed as a visual analogy to the Digit Span subtest and also includes forward and backwards span components. In the Forward Span component, the examiner points one-at-a-time to a series of spatial locations (demarked by blocks on a board). The participant must then point to the same spatial locations in the same order. In the backwards span component, the examiner again points to a series of spatial location, but the participant must now respond in the reverse order. As with the Digit Span subtest, the length of the sequence increases as the participant responds correctly.

#### 2.3.2. Inhibitory Control

A flanker visual filtering task was used to assess inhibitory control. The stimuli and procedure are identical to those employed in another recent study of inhibitory control in ASD [37]. In brief, participants were seated in front of a computer monitor and two large response buttons. They were asked to respond as quickly as possible based on the orientation of a centrally presented stimulus (e.g., “press the left button if the fish is facing left, and press the right button if the fish is facing right”). At the time of presentation, the target stimulus was closely flanked (<0.5°) to the left and right by distracting stimuli (i.e., additional fish). These stimuli were either compatible (i.e., facing the same direction) or incompatible (i.e., facing the opposite direction) with the target stimulus.

Together the stimuli subtended approximately 1° vertically and 10° horizontally (1.9° per fish). For each trial, stimuli were presented until a response was made or until more than 3000 ms elapsed. After an intertrial interval of 1500 ms, a new trial was presented.

If a child responded in less than 200 ms after presentation of the target (an anticipatory error), a brief tone followed by the message “early response” was presented. If a child failed to respond within 3000 ms (an inattentive error), a tone and “too slow” were presented. If a child responded by pressing the incorrect button (an accuracy error), a tone and “wrong response” were presented.

Children completed two practice blocks of 20 trials. In the first block, target stimuli were presented without flankers. In the second block, practice trials were identical to experimental trials (i.e., flankers were present). After practice, children completed 120 experimental trials, with 60 trials in each of the two conditions. Presentation was balanced such that all possible stimulus-flanker pairings were equally likely to occur. The conditions were mixed randomly. At intervals of 40 trials, children were offered a break.

Response time (RT) and error rates were recorded. Inhibitory control is assessed by comparing performance in the incompatible condition (in which participants must inhibit the conflicting flanker stimuli) and performance in the compatible condition.

#### 2.3.3. Task Switching

**Figure 1:**
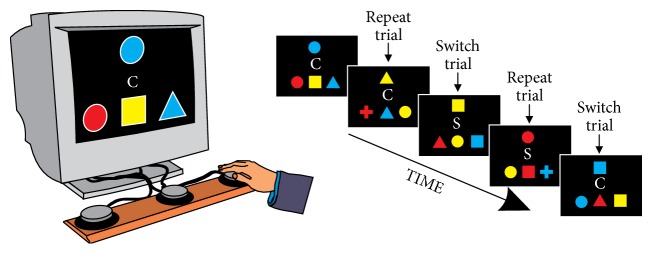
Illustration of the experimental apparatus and task conditions for the task switching test. Participants were instructed to match the top shape with one of the bottom three shapes. If the letter in the middle was a “C,” then they were to match based on color. If it was an “S,” then they were to match based on shape. [Note that the relative size of the stimuli has been enlarged for illustrative purposes.]

The experimental apparatus and task conditions are illustrated in Figure 1. Children were seated in front of a computer monitor and three large response buttons. On each trial, participants were shown a target stimulus at the top of the display and a row of three response stimuli near the bottom of the display. The target stimulus could be any of four different shapes (square, circle, cross, and triangle) presented in any of four different colors (red, green, blue, and yellow). One of the response stimuli shared the same shape as the target stimulus but not the same color. Another of the response stimuli shared the same color as the target stimulus but not the same shape. And the last response stimulus was a different shape and color than the target stimulus. The horizontal location (left, center, or right) of the different response stimuli varied randomly from trial to trial. A large response button was located below each of the three horizontal locations.

Concurrent with presentation of the aforementioned stimuli, a cue stimulus was presented at the center of the display. Participants were instructed that when they saw the “shape” cue (the letter “S” superimposed on a gray-colored hexagon shape), they should press the response button corresponding to the response stimulus that matched the target stimulus in shape. Similarly, when they saw the “color” cue (the letter “C” superimposed on a rainbow-colored diamond shape), they were to press the response button corresponding to the response stimulus that matched the target stimulus in color. RT and error rate were recorded. Following the participant's response and a short intertrial interval (50 ms), a new trial was presented.

Prior to beginning the task, participants were shown an illustration of both color and shape trials. Participants first completed a practice block of 24 trials of randomly intermixed color and shape trials. Next, 6 blocks of trials, each comprising 48 experimental trials, were administered. One experimental block included only color trials (the “pure color” block), and another block included only shape trials (the “pure shape” block). The remaining four blocks (“mixed” blocks) included an equal number of color and shape trials (24 of each) that were randomly intermixed. Prior to each block, the task instructions were reviewed, and participants were informed that the upcoming block would include color trials, shape trials, or a mix of both. The presentation order of the 6 experimental blocks varied randomly from participant to participant.

#### 2.3.4. Social Communication Abilities

The Social Responsiveness Scale (SRS; [63]) is a standardized, parent report, 65-item rating scale that measures social impairments associated with ASD across five domains: social awareness, social cognition, social communication, social motivation, and autistic mannerisms. A four-point Likert scale was used to rate items and scores were summed to form five subscales. A higher score reflected greater social impairment in that domain. *T*-scores derived from large-scale norming were provided in the scoring manual; however in order to better represent variations among very high scores, raw scores served as the unit of analysis in this study (consistent with other intervention research).

As noted above, the ASD participants in the present study were also concurrently enrolled in a larger efficacy study of the SCI-A program and their SRS outcome data has been reported in the prior study [22]. For comparison purposes, however, data is represented here as the primary measure of social competence improvement to be associated with any concurrent EF gains. The SRS was administered only for ASD/intervention participants; the non-ASD group did not receive the SRS.

## 3. Results

EF task data were available for all participants with the following exceptions: Due to technical problems, complete pre/postintervention data on the task switching test was unavailable for five participants with ASD. Data on the task switching test was also unavailable for the non-ASD comparison group. [Note that the non-ASD group data were originally collected as part of another (unpublished) study effort. However, given that the sole difference in study battery between the two groups related to the absence of the task switching test, we felt that the value of including their data for comparison purposes on the working memory and inhibition tasks outweighed any potential concerns.]

 For the inhibitory control and task switching tests, median response time (RT) for nonerror trials served as the primary dependent variable. Error rates were very low for both tasks (overall error rate for inhibitory and task switching tests = 2.9% and 5.7%, resp.) and thus were not considered further. For the current analyses, we employed mixed model analysis of variance (ANOVA) as the primary statistical model. ANOVA has been generally found to be robust to the assumption violations (e.g., assumptions of normality and homoscedasticity) that frequently arise with the analysis of RT data [64]. However, so as to further validate the present results, the analyses of EF performance were repeated utilizing a nonparametric statistical model (i.e., Mann–Whitney *U* test) that does not rely on such assumptions. The resulting pattern of statistically significant and nonsignificant findings was identical to that found with ANOVA. When applicable, effect sizes are reported in terms of Hedge's *g*, which provides a better estimate (in comparison to Cohen's *d*) when dealing with relatively smaller sample sizes. Descriptive statistics (mean, standard deviation, and range) for performance on each task are included in Table 3.

### 3.1. Working Memory

**Figure 2:**
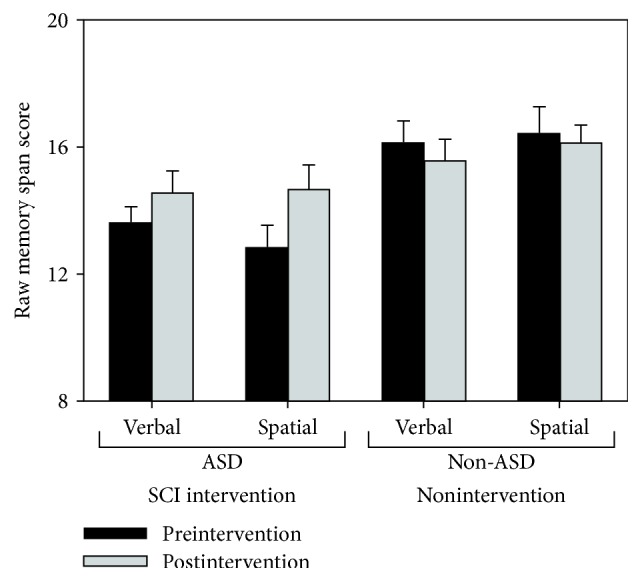
Mean raw scores for the working memory span tests, shown separately for each group (ASD and non-ASD), modality (verbal/digit and spatial), and timepoint (before and after intervention). Error bars represent standard error of the mean.

Mean raw scores for each working memory span test are shown in Figure 2. Data were entered into a 2 × 2 × 2 mixed model ANOVA with group (ASD and non-ASD) as a between-subjects factor, and modality (verbal and spatial) and timepoint (pre- and postintervention) as within-subject factors. There was no main effect of modality or timepoint [*F*(1,34) < 2.6, *p* > .12, *η*_*P*_^2^ < .07 in both instances]. However, a main effect of group was found, with the non-ASD group performing better overall than the ASD group [*F*(1,34) = 7.89, *p* = .008, *η*_*P*_^2^ = .19]. There was also a significant interaction between group and timepoint [*F*(1,34) = 9.05, *p* = .005, *η*_*P*_^2^ = .21]. No other interactions approached significance [*F*(1,34) < 1, *p* > .39, *η*_*P*_^2^ < .03 in all instances].

As can be seen in Figure 2, then interaction between group and timepoint was driven by the fact that the ASD intervention group showed improvements on the spatial subtest [*M*_PRE_ = 12.8; *M*_POST_ = 14.6; *t*(21) = 2.55, *p* = .02, *g* = .51] and the verbal (digit) subtest albeit to a slightly lesser degree [*M*_PRE_ = 13.6; *M*_POST_ = 14.6; *t*(21) = 1.70, *p* = .10, *g* = 0.33]. In contrast, the non-ASD group showed effectively no change in spatial or verbal working memory task performance over the same period of time [*t*(13) < 1, *p* > .41, *g* < .23 in both instances].

### 3.2. Inhibitory Control

**Figure 3:**
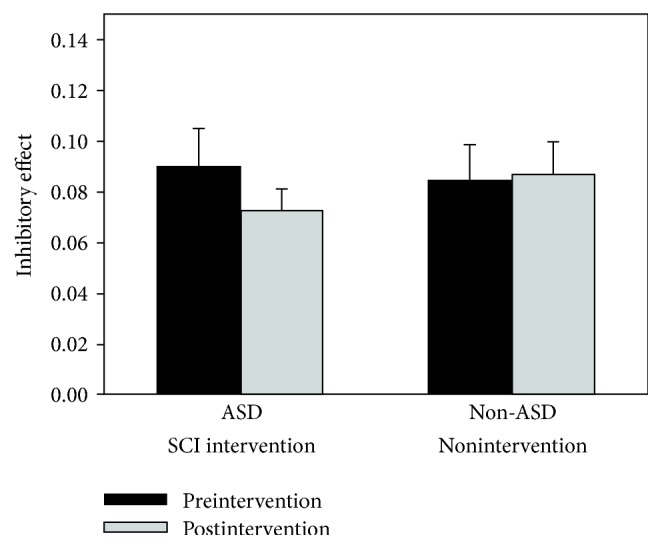
Mean inhibitory control effect [(RT_INCOMPATIBLE_ − RT_COMPATIBLE_)/RT_COMPATIBLE_] for the inhibitory control test, shown separately for each group (ASD and non-ASD) and timepoint (before and after intervention). Error bars represent standard error of the mean.

Inhibitory control data are shown in Figure 3. Potential intervention-related changes in the* inhibitory effect* or the additional time needed to respond on incompatible trial (i.e., a trial with flanking stimuli that are mapped to a competing response) as compared to a compatible trial (i.e., a trial with flanking stimuli that are mapped to the same response) were examined. Note that, in order to account for individual differences in general response speed, inhibitory effect was calculated as a proportion of the RT in the baseline compatible condition [(RT_INCOMPATIBLE_ − RT_COMPATIBLE_)/RT_COMPATIBLE_]. Resulting data were entered into a 2 × 2 mixed model ANOVA with group (ASD and non-ASD) as a between-subjects factor and timepoint (before and after intervention) as a within-subject factor.

No main effect of group or timepoint was found [*F*(1,33) < 1, *p* > .55, *η*_*P*_^2^ < .02 in both instances]. The interaction between group and timepoint also did not approach significance [*F*(1,33) < 1, *p* = .46, *η*_*P*_^2^ =.02]. No significant changes in inhibitory effect were observed for either the ASD intervention group [*M*_PRE_ = .090; *M*_POST_ = .073; *t*(20) = 1.08, *p* = .30, *g* = 0.31] or non-ASD group [*M*_PRE_ = .085; *M*_POST_ = .087; *t*(20) < 1, *p* = .92, *g* = .04].

### 3.3. Task Switching

#### 3.3.1. Switch Costs

**Figure 4:**
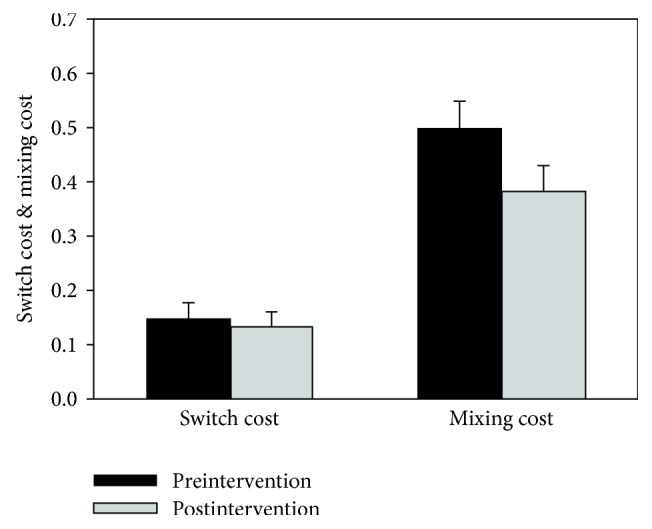
Mean switch cost [(RT_Switch_ − RT_Repeat_)/RT_Repeat_] and mixing cost [(RT_Mixed_ − RT_Pure_)/RT_Pure_] for the ASD intervention group on the task switching test, shown separately for each trial type (switch and repetition), block type (pure and mixed), and timepoint (before and after intervention). Error bars represent standard error of the mean.

Data for the task switching test are shown in Figure 4. Potential intervention-related changes in the* switch cost* or the additional time needed to respond on a switch trial (i.e., a trial in which the matching rule is different from that required on the previous trial) as compared to nonswitch/repetition trial (i.e., a trial in which the matching rule is the same as that required on the previous trial) within the mixed blocks were examined. In order to account for individual differences in general response speed, the switch cost effect was calculated as a proportion of the RT in the repetition trial condition [(RT_Switch_ − RT_Repeat_)/RT_Repeat_]. No significant pre/postintervention differences in switch cost were observed for the ASD group [*M*_PRE_ = .149; *M*_POST_ = .134; *t*(16) < 1, *p* = .62, *g* = .13]. [Note that, as explained earlier, comparison data from the non-ASD group were unfortunately unavailable for the task switching test.]

#### 3.3.2. Mixing Costs

Inclusion of pure blocks of trials (i.e., blocks of trials where the matching rule remained the same throughout the block) in the experimental design allowed for evaluation of another task-related phenomenon, namely, the* mixing cost*. The mixing cost reflects the observation that RT for repetition trials within mixed blocks (i.e., blocks also containing switch trials) tends to be slower than RT for repetition trials within pure trial blocks. It is generally believed that whereas switch costs (see above) reflect task switching ability, mixing costs may be better conceptualized as reflecting the added working memory demands associated with maintenance of two sets of task rules for the mixed blocks as compared to only one set for the pure blocks [31].

In order to account for individual differences in general response speed, the mixing cost effect was calculated as a proportion of the RT in the pure block condition [(RT_Mixed_ − RT_Pure_)/RT_Pure_]. A clear trend towards decreased mixing cost from pre- to postintervention was found [*M*_PRE_ = .50; *M*_POST_ = .38; *t*(16) = 1.97, *p* = .07, *g* = .57].

### 3.4. Relationship between Improvements in Executive Function and SRS Scores

As anticipated, participation in the SCI-A program was associated with improved social competence as reflected by significantly lower SRS raw scores at postintervention (*M*_POST_ = 87.6; Range: 59–118) as compared to preintervention (*M*_PRE_ = 106.1; Range: 71–144) [*t*(21) = 6.81, *p* < .001, *g* = 0.99]. No direct relationship was found between the observed intervention-related improvements in overall SRS scores and performance changes in the working memory (*r*_*s*_ = .09, *p* = .68), inhibitory control (*r*_*s*_ = .32, *p* = .16), or switching task (switch costs: *r*_*s*_ = .14, *p* = .54; mixing costs: *r*_*s*_ = .34, *p* = .13). Analysis of changes in SRS subscales (i.e., Social Awareness, Social Cognition, Social Communication, Social Motivation, and autistic mannerisms) and task performance yielded similar findings (*r*_*s*_ < .36, *p* > .09 in all instances).

## 4. Discussion

Whereas previous research on the SCI-A program has found significant improvements in measures of social competence and EF [22, 23], the measures utilized in these past studies have been largely limited to parent report questionnaires (e.g., BRIEF) and broad nonspecific measures of EF (e.g., TOPS). Consequently little is known regarding potential changes in specific EF component processes. By separately assessing working memory, inhibitory control, and cognitive flexibility at both pre- and postintervention, the current study was designed to further elucidate the nature of changes in EF functioning associated with participation in the SCI-A program.

Consistent with the aforementioned previous research, the present study found significant improvements in EF as measured by the present behavioral tasks. Specifically, intervention participants' performance on the working memory task improved significantly before to after intervention, with clear gains evident in spatial (nonverbal) working memory (*g* = 0.51). A nonsignificant trend towards improvement was also observed for verbal working memory performance (*g* = 0.33). At the same time, no such improvements were observed for the nonintervention comparison group suggesting that the performance gains of the intervention group are likely not attributable to practice and/or maturational factors. The finding of improved working memory performance for the intervention group is consistent with research with other clinical populations suggesting that working memory represents an aspect of cognition that may be malleable and responsive to intervention [65–67].

We also examined potential intervention-related changes in inhibitory control, specifically the ability to resist interference from visual distractors. As noted earlier, research has found that children with ASD are particularly impaired in this aspect of inhibitory control [36, 37]. Interestingly, we found no evidence of inhibitory control impairment in the present sample of ASD participants when compared to their non-ASD counterparts. It is important to note, however, that, in the aforementioned study by Christ et al. [37], they found a significant group by age interaction such that inhibitory control impairments were much more evident in their younger as compared to older participants. Within this context, it is possible that the age of the current participants was prohibitive (i.e., they were relatively too old) in terms of the manifestation of inhibitory difficulties.

With no impairment evident for the ASD group at baseline, it is not completely surprising that we also failed to find significant pre-to-post changes in inhibitory control ability for this group. It remains possible, had we targeted a younger cohort (whom would have presumably shown inhibitory impairment at baseline), that intervention-related improvements in inhibitory ability may have been observed as well. Indeed, even with no impairment at baseline in the present ASD sample, there was a nonsignificant trend (*g* = 0.31) towards improved inhibitory performance in this group following the intervention. Additional research is needed to further examine these possibilities and whether inhibitory ability may be responsive to intervention as well.

With regard to the switching task, we failed to observe intervention-related changes in our primary measure of cognitive flexibility/switching ability, namely,* switching costs* or the additional time needed to respond on a switch trial (i.e., a trial in which the matching rule is different from that required on the previous trial) as compared to nonswitch/repetition trial (i.e., a trial in which the matching rule is the same as that required on the previous trial). We did, however, find evidence of a nonsignificant trend (*g* = 0.57) towards intervention-related improvements in a secondary measure, namely,* mixing costs* or the additional time taken to respond when trials are presented within mixed blocks (i.e., blocks containing both “shape” and “color” matching trials) as compared to pure blocks (i.e., blocks containing “shape” or “color” matching trials but not both). Importantly, mixing costs are believed to not reflect switching ability per se but rather to reflect the added working memory demands associated with maintenance of two sets of task rules for the mixed blocks as compared to only one set for the pure blocks [31]. Within this context, the observed pre- to postintervention change in mixing cost may better conceptualized as an improvement in working memory performance rather than switching ability. Although speculative, the same neurocognitive processes underlying the improvements observed on the working memory span tests may also be responsible for this improvement as well.

As noted earlier, switching task data was unavailable for the nonintervention group. As such, even though there was no evidence of practice effects for either of the other two tasks, we cannot fully rule out the possibility that practice and/or maturational factors may have contributed to the* mixing costs* improvement observed on the switching task. Additional research is needed to rule out this possibility and to further explore the relationship between the improvements observed on the working memory span tasks and those on the switching task.

The present findings add to the growing evidence of improvements in EF in children with ASD participating in social skills training that emphasizes the use of EF skills such as problem solving in conversation [22, 23]. This line of research further supports the use of curricula such as SCI-A that integrates EF skills and application within its delivery. Furthermore, whereas these earlier studies relied almost exclusively on parent report measures (e.g., the BRIEF), the current study utilized performance-based behavioral measures, which are generally accepted to be less susceptible to informant bias. The present methodology also allowed for more precise characterization of the observed improvements such that we found that EF improvements associated with participation in the SCI program were most apparent on working memory-related measures (as compared to inhibitory control or switching). Future research utilizing a similarly approach may prove useful in also refining our understanding intervention-related changes in non-EF processes as well (e.g., emotion recognition, pragmatic language). In addition, by identifying those contributing processes which are most malleable, research such as this may inform future intervention design and planning.

### 4.1. Additional Limitations and Future Directions

Although both social competence and executive function performance improved with SCI-A participation, we failed to find a direct correlation between the measures of the two constructs within the current study. A number of factors may have contributed to this finding (or lack thereof). For example, whereas the present sample size was comparable to that utilized in several past social competence- and EF-focused ASD intervention studies [10, 21, 22, 68, 69] and appeared sufficient to detect group-related changes in the aforementioned constructs, it was likely undersized/underpowered to fully elucidate the relationship among individual differences in these constructs. Additional research with a larger sample size is also needed to confirm the generalizability of the present findings to the broader population as well as evaluate the extent to which the observed differences may be “clinically meaningful.”

Another potential factor relates to differences in the nature of the measures used to assess EF and social competence. Executive function was assessed using performance-based behavioral measures administered within a laboratory setting. In contrast, social competence was assessed using a report-based measure (i.e., the SRS) which focused on the manifestation of social competence ability within the participants' everyday environment. In light of previous research demonstrating only a modest correlation between performance-based and report-based measures even when assessing the same construct (e.g., EF: [70, 71]), the present lack of correlation between performance- and report-based measures of EF and social competence, respectively, is not all too surprising and is not necessarily indicative of a lack of relationship between the two underlying constructs.

The aforementioned methodological mismatch reflects a larger issue within the field, namely, a paucity of performance-based measures of social competence that are well-established and have been demonstrated to be psychometrically sound. The continued development and validation of such measures are essential for future efforts to fully elucidate the nature of the relationship between social competence and other cognitive constructs such as EF.

On a related note, the present tasks were designed to assess core EF component processes in relative isolation while placing minimal demand on other social-related cognitive processes such as language and affect processing. This approach may be viewed as a double-edged sword. On one hand, it allows for isolation of individual component processes. On the other, implementation of executive function in everyday life typically involves placing concurrent demands on multiple EF and non-EF processes. It is possible that a more robust effect would have been observed on more complex multidemand tasks, for example, one aimed at assessing EF in a social context (i.e., the focus of the present intervention).

Lastly, it is worth considering whether or not undiagnosed secondary symptoms related to ADHD or another comorbid condition may have contributed to the present findings. This is particularly relevant given the relatively high prevalence rate of children with comorbid ASD and ADHD [72–74]. Research suggests that these two disorders are each associated with distinct profiles of executive dysfunction [32, 47]. For example, whereas ADHD is associated with significant impairments in inhibitory control [75], findings on inhibitory control in ASD (in absence of ADHD) are much less clear [35]. In addition, while both ASD and ADHD are associated with working memory impairment, the severity of impairment appears exacerbated in individuals with comorbid ASD and ADHD [33]. In terms of the present study, the lack of a significant inhibitory impairment at baseline runs contrary to expectations if there had been extensive ADHD comorbidity among the study's ASD participants. Regardless, additional research is necessary to examine whether the presence/absence of comorbid ADHD symptomatology may influence the effectiveness of the SCI-A program for improving not only social competence ability but also underlying core EF processes such as working memory.

In the present study, intervention targeting social competence and the application of EF skills within a social context appeared to result in generalized benefits for performance on more basic nonsocial EF tasks. This finding is admittedly unusual. Research in this area typically centers on the somewhat reverse of this phenomenon, namely, how focused training (on EF or another particular area of cognition) leads to improvements in a more general area such as social competence. As such, if taken in isolation, the present results might be dismissed as spurious – particularly given the relatively modest effect sizes observed (*g* = 0.33–0.57). However, the current findings are only the latest in a growing body of evidence [22, 23] supporting the possibility of generalized EF improvements in SCI-A intervention participants. Taken together with these past studies, the present results raise a number of interesting questions about the dynamic relationship between social competence ability and underlying cognitive processes such as EF, questions that necessitate additional research.

As detailed earlier, the SCI-A program is designed for children who fit a specific phenotype (i.e., adolescent with HFA) and specifically targets EF as a critical factor in social competence impairments. Within this context, additional research is needed to evaluate to what extent the presently observed gains in EF performance may translate to other age ranges, levels of symptom severity, and variations of the SCI program (e.g., SCI-School: [23]; SCI-Elementary: [68]) as well as other social skills interventions.

### 4.2. Conclusions and Summary

The present results are consistent with previous reports [22, 23, 68] of generalized improvements in EF associated with participation in the SCI program. By using behavioral tests to separately evaluate core components of EF, we were able to more precisely characterize the nature of the changes in these cognitive processes. Specifically, the findings suggest that participation in the SCI program is associated with improvements in working memory performance, particularly spatial working memory. Working memory (both spatial and verbal) would seem to play a critical role in several aspects of proficient social communication such as turn taking in conversations (i.e., the ability to follow the flow of a conversation while concurrently preparing one's own contribution to the conversation), and identification of social cues and subsequent implementation and maintenance of appropriate metacognitive strategies to enhance a given social interaction. Additional research is needed to fully understand how EF improvements such as those observed in the present study may relate to improved social competence.

## Figures and Tables

**Table 1:** Sample characteristics.

Variable	ASD intervention group (*n* = 22)		Non-ASD group (*n* = 14)	
	*M* (SD)	Range	*M* (SD)	Range
Age (years)	12.3 (1.1)	10.8–14.7	12.8 (0.9)	11.0–14.5
Gender (M/F)	22/0		22/0	
FSIQ	100.5 (13.1)	78–130	103.4 (7.2)	81–111
Parent-report SRS (at baseline)				
Raw score	106.1 (18.8)	71–144	—	—
*T* score	84.6 (9.0)	68–103	—	—
ADI-R (*n* = 18)^*∗*^				
A (social interaction)	15.2 (6.9)	3–29	—	—
B (communication)	13.4 (5.4)	5–21	—	—
C (restricted/repetitive behavior)	4.7 (2.4)	1–10	—	—
ADOS-original algorithm (*n* = 7)^*∗*^				
Communication	1.9 (0.7)	1–3	—	—
Social interaction	6.6 (3.3)	0–10	—	—
ADOS-revised algorithm (*n* = 8)^*∗*^				
Social effect	7.4 (2.5)	4–11	—	—
Restricted, repetitive behavior	2.6 (1.7)	0–5	—	—

^*∗*^Note that a subset of children (*n* = 10) received both the ADI-R and ADOS.

**Table 2:** Association of SCI-A curricular units and executive functions.

SCI-A unit	Curricular content	Executive function^*∗*^
(1) Recognizing facial expressions	Visual recognition of key facial features Displaying facial expressions Strategies for scanning facial features to read emotions	

(2) Sharing ideas	Speaker skills: gaining attention, staying on topic, sharing the main idea, appropriate eye contact/body proximity/volume Listener skills: appropriate eye contact/body proximity	*Working Memory:* staying on topic *Inhibitory Control:* sharing only the main idea/not sharing irrelevant information; avoiding interrupting others

(3) Turn taking in conversation	Conversational reciprocity Using questions and comments Transitioning in/out of conversations	*Inhibitory Control:* avoid interrupting others *Working Memory:* staying on topic, building off another person's comments *Cognitive Flexibility:* switching/transitioning topics

(4) Feelings and emotions	Understand emotional range/variance/intensity Self-control and emotion regulation Using context to understand others' emotions/perspective taking	

(5) Problem solving	Identify components of problems (who, what) Generate and evaluate possible solutions Collaborate with others to solve problems	*Inhibitory Control:* use appropriate conversational skills to collaborate *Working Memory:* holding the ideas of others to collaborative problem solving *Cognitive Flexibility:* generate alternate solutions

^*∗*^Consistent with EF being only one of several interrelated constructs that contribute to social competence, the content in any given unit involves a combination of EF and other processes (e.g., social reciprocity, and pragmatic language).

**Table 3:** Descriptive statistics for performance on each task.

Variable	ASD intervention group				Non-ASD group			
	Timepoint 1		Timepoint 2		Timepoint 1		Timepoint 2	
	*M* (SD)	Range	*M* (SD)	Range	*M* (SD)	Range	*M* (SD)	Range
Working memory measure								
Spatial subtest	12.8 (3.3)^*∗*^	6–19	14.6 (3.7)^*∗*^	7–22	16.4 (3.1)	12–21	16.1 (2.0)	12–20
Verbal (digit) subtest	13.6 (2.4)	9–18	14.6 (3.3)	7–22	16.1 (2.5)	13–23	15.6 (2.5)	12–21
Combined (spatial + verbal)	26.4 (4.7)^*∗*^	17–33	29.2 (5.8)^*∗*^	14–40	32.6 (4.3)	27–40	31.7 (3.8)	26–40
Inhibitory control measure								
Inhibitory effect	.09 (.06)	−.03–.23	.07 (.04)	−.01–.12	.08 (.05)	.00–.18	.09 (.05)	.00–.16
Task switching measure								
Switching costs	.18 (.17)	−.02–.72	.15 (.13)	−.01–.42	—	—	—	—
Mixing costs	.54 (.24)	.26–1.21	.40 (.20)	.08–.82	—	—	—	—

^*∗*^Effect of Timepoint, *p* < .05.
